# Fluorescent Nanocrystals Reveal Regulated Portals of Entry into and Between the Cells of *Hydra*

**DOI:** 10.1371/journal.pone.0007698

**Published:** 2009-11-02

**Authors:** Claudia Tortiglione, Alessandra Quarta, Maria Ada Malvindi, Angela Tino, Teresa Pellegrino

**Affiliations:** 1 Istituto di Cibernetica “E Caianiello”, Consiglio Nazionale delle Ricerche (CNR), Pozzuoli, Italy; 2 Fondazione Istituto Italiano di Tecnologia, Genova, Italy; 3 National Nanotechnology Laboratory of CNR-INFM, Unità di ricerca IIT and Scuola Superiore ISUFI, Lecce, Italy; Johns Hopkins School of Medicine, United States of America

## Abstract

Initially viewed as innovative carriers for biomedical applications, with unique photophysical properties and great versatility to be decorated at their surface with suitable molecules, nanoparticles can also play active roles in mediating biological effects, suggesting the need to deeply investigate the mechanisms underlying cell-nanoparticle interaction and to identify the molecular players. Here we show that the cell uptake of fluorescent CdSe/CdS quantum rods (QRs) by *Hydra vulgaris*, a simple model organism at the base of metazoan evolution, can be tuned by modifying nanoparticle surface charge. At acidic pH, amino-PEG coated QRs, showing positive surface charge, are actively internalized by tentacle and body ectodermal cells, while negatively charged nanoparticles are not uptaken. In order to identify the molecular factors underlying QR uptake at acidic pH, we provide functional evidence of annexins involvement and explain the QR uptake as the combined result of QR positive charge and annexin membrane insertion. Moreover, tracking QR labelled cells during development and regeneration allowed us to uncover novel intercellular trafficking and cell dynamics underlying the remarkable plasticity of this ancient organism.

## Introduction

The plasma membrane is a dynamic structure regulating the entry and the exit of small and large molecules into the cell cytoplasm. Several mechanisms underlie particle internalization into transport vesicles derived from the plasma membrane. Although generally termed as “endocytosis”, they encompass different regulated endocytic pathways with regard to the nature of the cargo (receptor, ligand, lipids), the size of endocytic vesicle and the mechanism of vesicle formation [1]. The complexity of the molecular interactions underlying the endocytosis suggests that a great evolutionary effort has been spent to regulate the cellular response to a variety of different environmental stimuli. In multicellular organisms the endocytic and secretory pathways evolved to control all aspects of cell physiology and intercellular communication (neurotransmission, immune response, development, hormone-mediated signal transduction). The successful use in biology and medicine of functional nanoparticles and nanodevices based on innovative biomaterials, introduced in this scenery new classes of compounds, variable in size (from 2 to 100 nm), chemical composition (gold, cadmium telluride, cadmium selenide, iron oxide) and physical properties (charge, spectral profile, colloidal stability, magnetism). Thus, the specific interactions of these new biomaterials with cell membranes needs to be carefully investigated. Presentation of chemical information at the same size scale as that of cell surface receptor may potentially interfere with cellular processes, eliciting undesired responses, such as cell uptake, sequestration in endosomal/lysosomal compartments, or activation of signalling cascade pathways. In this frame, before employing any nanostructure for biological imaging, diagnostic and therapeutic application, the interfacing bio-non bio must be evaluated.

Due to their superior brightness, higher photostability and narrower spectral emission compared to conventional organic fluorophores, spherical and rod shaped fluorescent semiconductor nanocrystals, also known as Quantum dots (QDs) or Quantum rods (QRs), are more and more used to probe biomolecular interaction in living cells, to study intracellular processes at single-molecule level, high resolution cellular imaging, as well as for long-term *in vivo* observation of cell trafficking, tumor targeting, and diagnostic [2]–[9].

Because of the variety of well established or new published nanocrystal synthesis, solubilization and functionalization protocols, which found our group deeply involved [10]–[15] and the diverse experimental systems (cell lines, tissue or animals) used to test them, not general rules exist to predict the interaction between nanocrystals and the targeted cell membrane and the effect of long-term exposure. Evidences are cumulating that nanoparticles play active roles even in the absence of specific ligands and that factors such as size and charge are crucial for activation of cell responses [16], internalization [17], [18], and intracellular trafficking [19]–[21].

Generally, live studies in higher vertebrates relying on the injection of nanoparticles into the bloodstream are limited by the opsonization process, namely the coating of nanoparticle surface by components of the circulation, such as plasma proteins. This process renders the particle recognisable by the reticulo-endothelial system (RES), which provides to their phagocytosis. Longer circulation times have been allowed by coating nanoparticles with dense brushes of polymers, such as polyethyleneglycol (PEG), polyethylene oxide (PEO) [22], [23], which generally enhances colloidal stability of nanoparticles in biological melieu. Alternative water solubilization routes or colloidal stabilizating coatings are also being proposed in order to avoid potential cytotoxic issues [11], [13], [24]. Despite all efforts, however, complete evasion of the RES by these coated nanoparticles has not yet been possible as well as aspecific uptake from non phagocytic cells, and alternative *in vivo* systems to study the cellular response to unfunctionalised nanoparticles are needed [25], [26].

At the base of metazoan evolution the freshwater *Hydra vulgaris* has been shown an amenable system to study the interaction between nanoparticles and living systems [27], [28]. The dipoblastic polyp is composed of two epithelial cell layers (an inner endoderm and an outer ectoderm facing the low ionic strength medium) with few interspersed specialised cell types, a neuronal net controlling functions and physiology. This structural complexity, simpler than vertebrates, with central nervous system and specialized organs, but much complex compared to cultured cells, makes *Hydra* comparable to a living tissue which cells and distant regions are physiologically connected. In a previous work, exposing living polyps to QRs added in the medium, resulted in the induction of an unexpected behavioural response, controlled by tentacle's neurons [28]. This peculiar response, to rod but not to spherical shaped nanoparticles, lead to the hypothesis that electrical properties of nanoparticles may underlie neuronal activation, showing the great potential of using this simple organism to reveal the impact of nanoparticle physical properties on animal physiology and cell biology.

In this paper, using this model system, we assess *in vivo* the relationship between amino-PEG coated CdSe/CdS core/shell QRs and cell uptake. By tuning the number of amino-PEG molecules attached at the rod surface and thus by manipulating the resulting surface charge at different pHs, we tuned the capability of *Hydra* ectodermal cells to uptake QRs, from very high at acidic pH to zero at neutral pH. Only under acidic conditions *Hydra* ectodermal cells bind and sequester into cytoplasmic granular structures positively charged QRs, while at neutral pH this does not occur. In the attempt to identify the molecular targets underlying QRs internalization at acidic pH, we tested the involvement of annexin XII (ANX), a *Hydra* protein belonging to the annexins superfamily [29], [30], able to insert into lipidic membranes and to form ion channels at acidic, but not neutral pH [31]. As *Hydra* treatment with anti-ANX antibody prevents QRs uptake, we show the involvement of ANX in the QRs uptake at acidic pH, and provide a first functional role for annexin XII *in vivo*. Moreover, due to the extreme photostability of the inorganic nanoparticles, tracking QR labelled ectodermal cells over long periods of times led to the discovery of new migration dynamics and intercellular trafficking events in the tentacles and subhypostomal region, monitored and characterised both in normal growth and regeneration conditions.

## Results

### QR uptake by living Hydra can be controlled by manipulating surface charge

**Figure 1:**
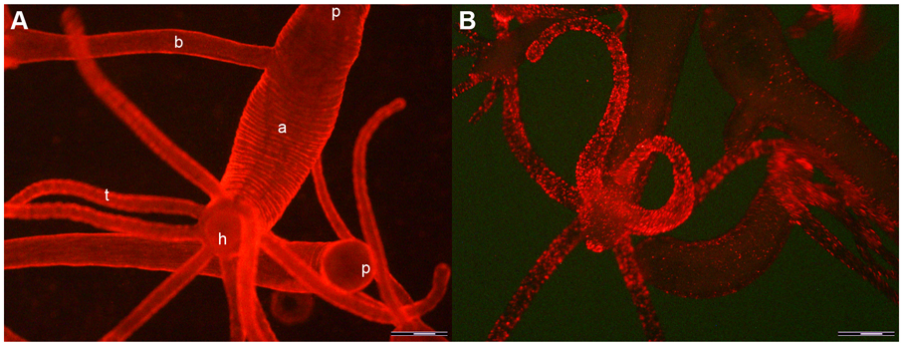
*In vivo* fluorescence imaging of *Hydra vulgaris* exposed to QRs for different times. A) *In vivo* image of two *Hydra*, 30 minutes p.i.: QR red fluorescence labels uniformly all body regions, from the tentacles (t) to the peduncle (p) located in the upper part of the image. In this picture an adult (a) with a bud (b) on the left side turns towards the camera the hypostome (h), surrounded by a ring of tentacles. A second *Hydra* is placed horizontally below, bending the peduncle round the camera. B) *In vivo* image of different polyps, 2 h p.i. with QRs. In the foreground a *Hydra* turns the hypostome toward the bottom. A strong punctuated fluorescence labels the mouth, the tentacles and at a lower extent the animal body. Scale bar 500 µm.

**Figure 2:**
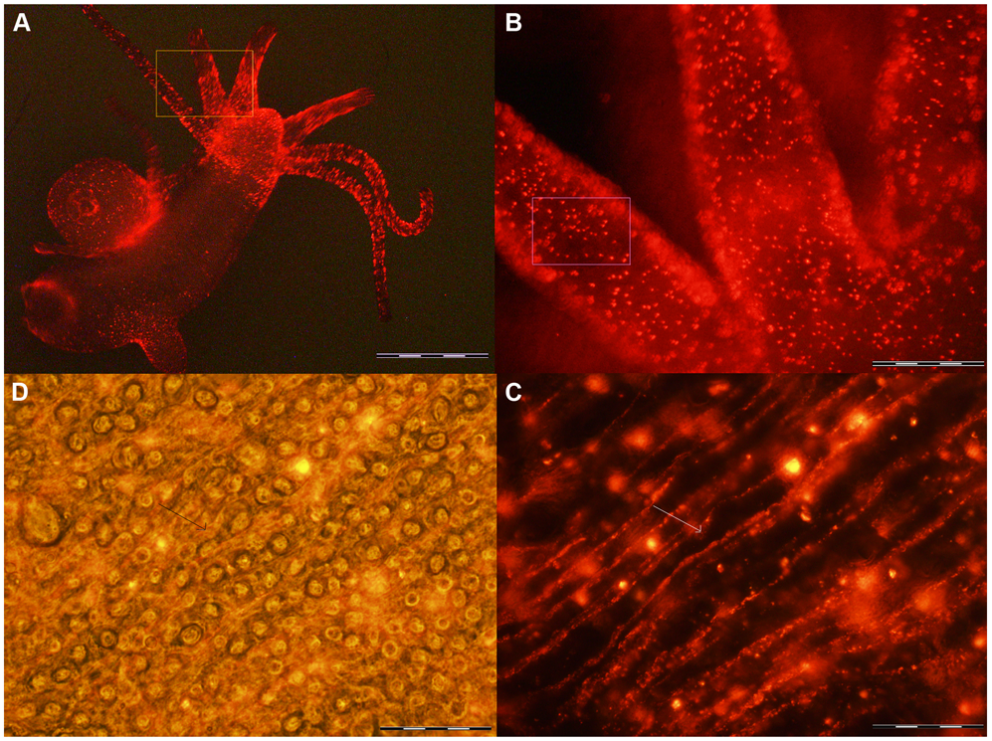
Tissue distribution of QR labelled cells. A) *In vivo* imaging of a whole animal, incubated with QRs in SolHy, at pH 4, for 2 h, washed and observed by fluorescence microscopy. QR staining is distributed on tentacles, hypostome and peduncle. The region within the orange frame is shown at higher magnification in B) punctuated fluorescence is uniformly distributed over the tentacles. Regions out of focus belong to different planar levels. C) Image showing a region within the pink inset of B, at higher magnification. Single animals were put on a microscope concave glass slide, with a drop of SolHy and observed under fluorescence or phase contrast mode (D). QRs label cell membranes of battery cells (arrows), but are also compacted into cytoplasmic granule-like structures, which do not colocalize with nematocytes, as evident in the bright field-fluorescence merged image (D). The round structures represent the numerous nematocytes (desmonemes, stenotheles and izorhiza) embedded in the cytoplasm of the battery cells. Scale bars: 1 mm in A, 200 µm in B, 20 µm in C e D.

**Figure 3:**
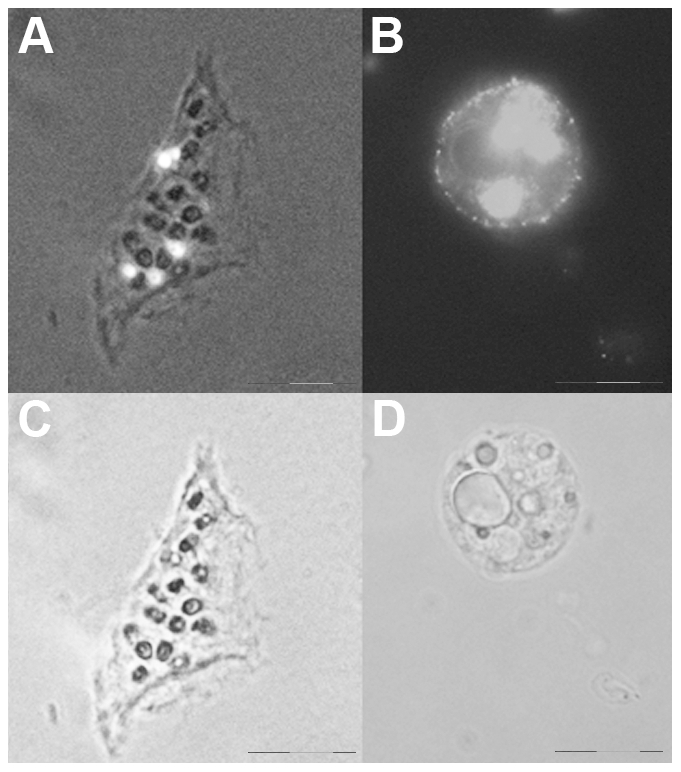
Fluorescence imaging of fixed and viable single cells obtained from whole treated animals. Whole animals were incubated with QRs for 4 h, extensively washed and then dissociated into a suspension of fixed (A) or viable (B) cells and imaged using fluorescence or/and phase contrast microscopy. A) Picture of a battery cells containing embedded in the cytoplasm numerous nematocytes (black circles). QRs appear compacted into at least 5 granules (white spots). The image is an optical merging of phase contrast and fluorescence microscopy. The colour information as been eliminated using the software Cell F (Olympus) B) Fluorescence image of a living labelled cell, showing large fluorescent vesicles inside the cytoplasm, and QRs labelling also on the cell membrane. C) and D) are bright field images of A and B, respectively. Scale bars = 20 µm.

**Figure 4:**
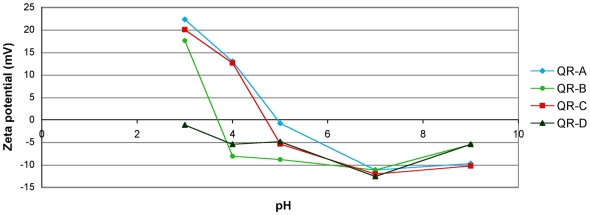
Zeta potential of the amino-PEG coated QRs as a function of pH. Zeta potential is widely used to quantify the electrical charge in colloidal systems [37]. While at neutral pH all amino-PEG QR samples display negative zeta potential, at pH 4 only QR-A (blu line) and QR-C (red line) presented positive values, showing the involvement of QR positive charge in the cell uptake. Measurements were performed in SolHy.

In our previous works, incubating living *Hydra* at neutral pH with CdSe/ZnS core/shell QDs, or with CdSe/CdS QRs, did not result in nanocrystals internalization into *Hydra* cells [27], [28]. With the aim to elucidate the physicochemical and molecular factors underlying the interaction between QRs and *Hydra* cells, respectively, we challenged living polyps with QRs at different pHs, modifying both players of the bio-non bio interaction: the surface charge of the nanorod at one side, and the biophysical properties of the cell membranes at the other side. Asymmetrical CdSe/CdS core/shell QRs were synthesized according to a recently reported procedure [32]. Rods sized 35×4 nm were transferred from chloroform into water by wrapping them by an amphiphilic polymer [14] and then by linking diamino terminated PEG molecules to their surface [33] via the EDC crosslinker chemistry [34]. The resulting highly fluorescent amino-PEG coated QRs (Figure S1), named QR-A, were added to the medium of living polyps under different ionic conditions and pH values. While in physiological medium or at slightly lower pH values (pH 6 and pH 5) polyps were not visible by fluorescence microscopy, unless a faint green autoflorescence signal, an intense bright and red labelling was obtained at pH 4. As shown in Table 1, QR cell uptake occurred only at acidic pH, in a Ca^2+^ independent way, while was never detected at neutral pH, confirming our previous finding [28]. Membranes of ectodermal cells all over the body appear intensively labelled, from tentacle tips to emerging bud and foot region (Figure 1A). After 2 h of continuous incubation the uniform surface labelling becomes compacted into distinct structures located on the tentacles, around the mouth and to a lower extent on the gastric region (Figure 1B, 2A). At higher magnification, the strong punctuated fluorescence appears distributed both intracellularly and on membranes of battery cells (Figure 2B, 2C), the tentacle specific complexes composed of ectodermal cells embedding numerous nematocytes (the stinging cells used by the polyp for prey capture). The same region observed under bright field shows that the fluorescence is not located within nematocytes (Figure 2D). Analysis of single viable or fixed cells obtained from dissociation and maceration procedures [35], [36], respectively, confirmed the intracellular localization of QRs (Figure 3). The use of both procedures to examine single cell suspension led to detection of membrane labelling only in living dissociated cells, and not in fixed cells, either due to experimental artefacts, or to a fast effect of the chemicals used for fixation, perhaps pH dependent, on membrane trafficking. In order to test whether this behaviour was general for CdSe/CdS nanorods, or dependent from specific properties of the sample employed (QR-A), we performed the same assay using CdSe/CdS nanocrystals coming from different synthesis, and presenting different sizes (Figure S1 and Table S1). Results, shown in Table 1, indicate that QR samples displayed different behaviours with respect to the uptaking process. An estimation of their surface charge was achieved by zeta potential measurements, a method widely used to quantify the electrokinetic potential in colloidal systems [37]. The graph of Figure 4 revealed that nanocrystal samples producing similar *in vivo* effects were characterized by the same zeta potential-pH dependence profile. QRs uptaken by *Hydra* cells (QR-A, QR-C) show negative zeta potentials at neutral and basic pHs while, at pH 4, they assume positive values. By contrast, QR-B and QR-D, that *in vivo* are not internalized, present at pH 4 negative zeta potential values, indicating that the effective parameter that influences the animal uptake is even independent on shape of the nanoparticle but is rather dependent on its surface charge.

**Table 1:** Effect of Calcium and pH on QR uptake.

QR	Size (nm)	SolHy pH 7	SolHy pH 4	Ca^2+^free pH 7	Ca^2+^ free pH 4
QR-A	35×4	−	+	−	+
QR-B	35×4	−	−	−	−
QR-C	20×5	−	+	−	+
QR-D	20×20	−	−	−	−

QRs of various shapes and sizes were supplied to groups of living polyps (10 animals/well) in SolHy at the indicated pHs. Living *Hydra* were incubated for at least 2 h with QRs, then they were thoroughly washed and observed under fluorescence stereomicroscope/microscope. The presence of QR internalization is indicated as plus (+), while the absence as a minus (−). QR uptake occurs in a Ca^2+^ independent manner and only at acidic pH.

### QR uptake depends on the charge of surface coating

**Figure 5:**
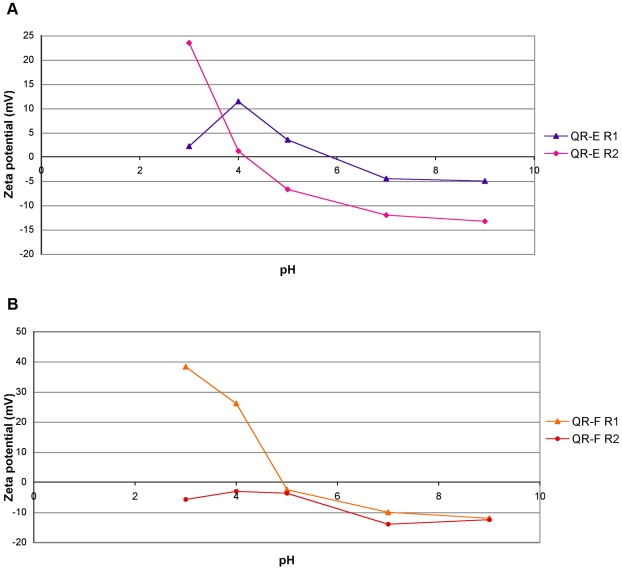
Zeta potential of QRs coated with different amount of amino-PEG as a function of pH. The zeta potential-pH profiles of QRs samples coated with different amounts of amino-PEG show the positive values of both QR-R1 type at pH 4, due to protonation of the amino and of the carboxy groups at the QR surface. The surface positive charge influences the electrokinetics of the colloidal particles, promoting their binding to cell membranes and intracellular uptake. Measurements were performed in SolHy.

As the charge properties of QRs are determined by the nature of the functional groups at the nanocrystal surface, to correlate the surface chemistry of QRs to the cell uptaking behaviour observed, a simple experiment was carried out. Polymer coated QRs (QR-E and QR-F) of two different sizes (15×3 nm and 35×4 nm) were decorated with two different amounts of amino-PEG molecules. The amount of diamino-PEG to be attached at the nanocrystal surface was carefully chosen in order to switch the net surface charge of the same nanoparticle from negative to positive at pH 4 (see Table S2) and confirmed by gel electrophoresis (Figure S2). In Figure 5, zeta potential-pH profiles of these QR conjugates with high (-R1) or low (-R2) amounts of diamino-PEG are reported. The zeta potential values indicate at pH 4 positive surface charges for nanocrystals functionalized with high amount of amino-PEG (QR-R1), while by contrast, the same QR functionalized with low amounts of diamino-PEG at pH 4 present neutral (QR-E R2) or negative (QR-F R2) charges. The *in vivo* assay on *Hydra*, at the different pHs, confirmed that only QR-R1 nanorods entered *Hydra* ectodermal cells at pH 4, while QR-R2 did not (Table 2). This behaviour is tentatively assigned to the electrostatic interaction between the positive surface charges of the nanoparticles and the negative charged cell membranes. These data are in line with observations of other groups that have tested different types of inorganic nanoparticles in diverse biological systems [24], [38], [39].

**Table 2:** Effect of surface functionalization on QR uptake.

QR	Size (nm)	SolHy pH 7	SolHy pH 4
QR-E R1	15×3	−	+
QR-E R2	15×3	−	−
QR-F R1	35×4	−	+
QR-F R2	35×4	−	−

CdSe/CdS core/shell QRs were water transferred by means of an amphiphilic polymer and were stabilised by addition of high (R1) or low (R2) amount of diamino-PEG molecules to their surface via the EDC crosslinker chemisty (see Materials and Methods). Living *Hydra* were incubated in culture solution (SolHy) at pH 4 or pH 7 in presence of the indicated nanorod. QR-R1 conjugated with high amount of PEG, were uptaken by *Hydra* ectodermal cells at pH 4, while QR-R2 coated with less molecules were not.

### Cell tracking of QR labelled cells reveals interepithelial dynamics

**Figure 6:**
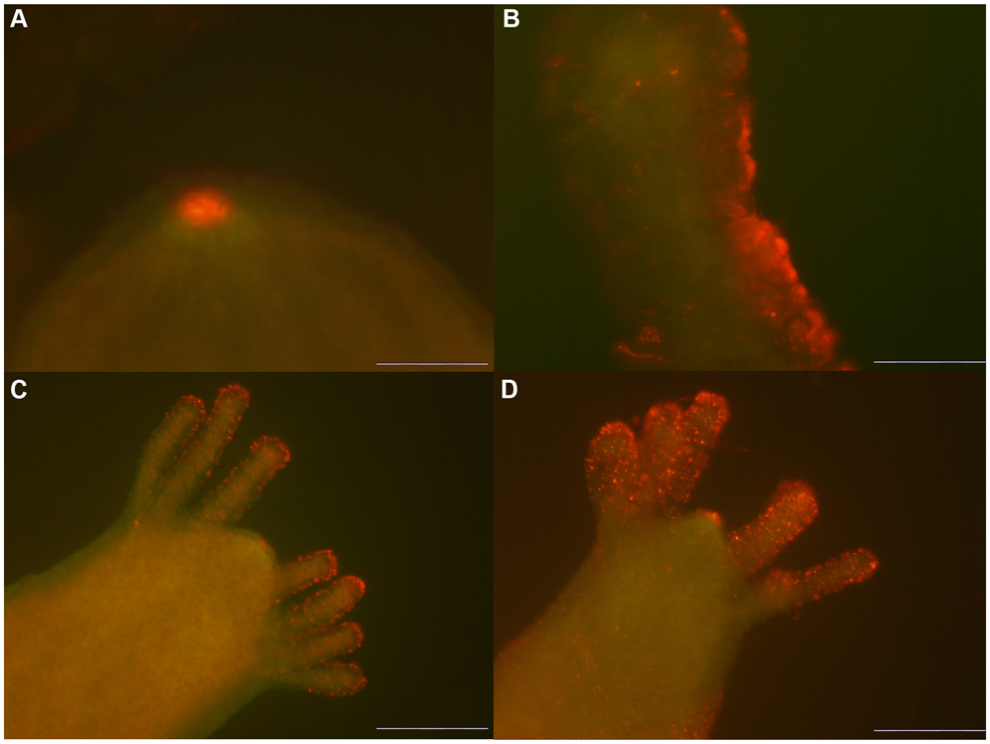
Time course of QR uptake. *Hydra* were challenged with QRs for periods ranging from 10 to 30 minutes, then extensively washed, fixed and mounted on microscope slides. Fixation treatment enhances the green colour due to tissue autofluorescence compared to *in vivo* imaging. 10 minutes p.i. (A) QRs are selectively uptaken by mouth cells located on the hypostome tip. On the tentacles (B) QR fluorescence appears uniformly dispersed inside battery cells, along the entire tentacle length. 20 minutes post treatment (C) the labelling pattern appears as granules located on the hypostome and the tentacles, whose number increases as incubation proceeds (D). Scale bar 50 µm in A, B, and 200 µm in C, D.

**Figure 7:**
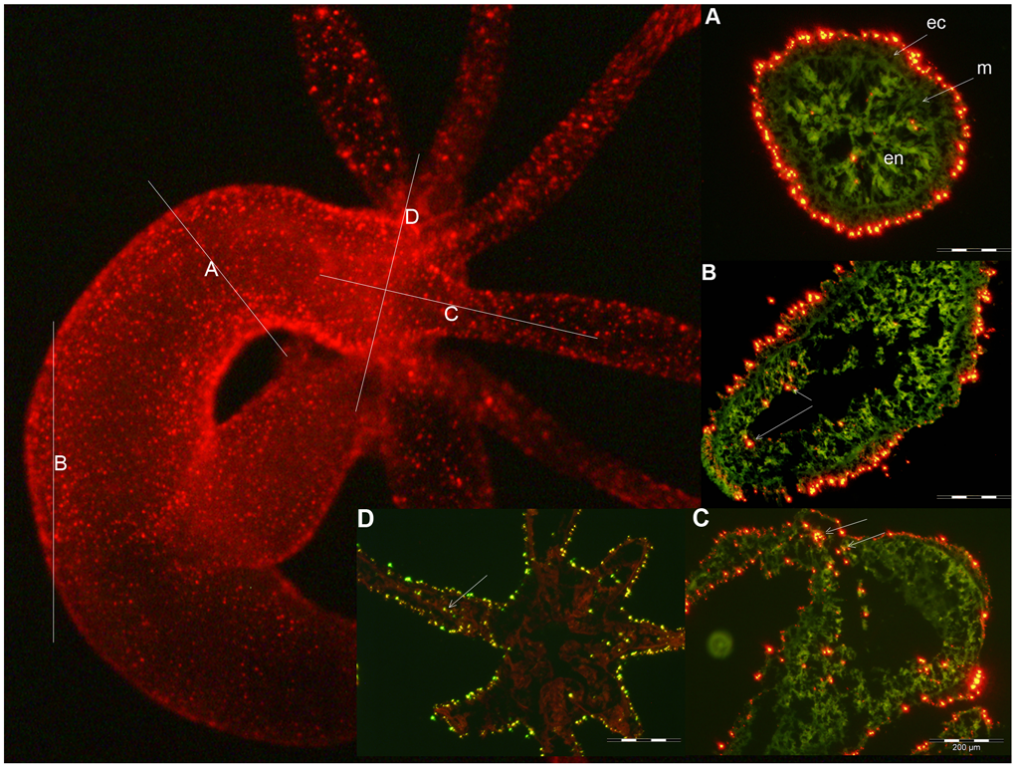
Cellular localization of QRs in *Hydra* tissue sections. Intact *Hydra* were treated with QRs at acidic pH for 4 h, and *in vivo* imaged 24 h later (left panel). On this image the orientations of sectioning planes (A, B, C and D) used for sectioning are shown as dotted lines, and on the right panels the corresponding tissue sections are reported. The green colour is due to tissue autofluorescence, while the red staining indicate the QR presence. Endodermal cells (en) are separated from ectodermal cells (ec) by an extracellular matrix, the mesoglea (m), indicated by the arrows. A) fluorescence image of a cross section obtained at the level of line A. QRs are located in the apical part of ectodermal cells. Some endodermal cells appear also fluorescent. B) longitudinal section at level of the gastric cavity showing heavy peripheral staining of ectodermal cells and also some fluorescent cells located into the endoderm (arrows) C) longitudinal section at the hypostomal level showing uniform ectodermal staining and endodermal QR localization within the tentacle emerging zone and the hypostome (arrows) D) section at the hypostomal level along a plane perpendicular to C. The tissue red colour is due to a toluidine staining used to contrast bright field imaging (not shown). The arrow shows QRs aligned in the tentacle endoderm.

**Figure 8:**
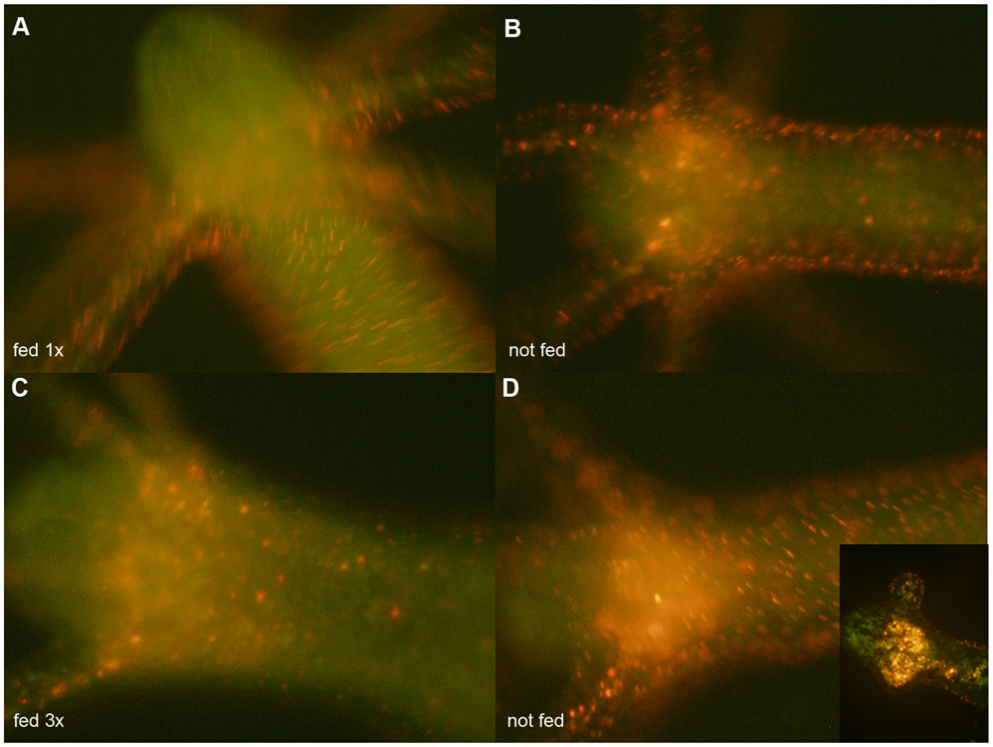
QR tracking under feeding and starvation regimes. Labelled polyps were cultured under normal feeding regimes (A and C), or kept under starvation condition (B and D) for the corresponding times. The first feeding started 24 h post treatment, and the following ones carried out every other days as detailed in the Materials and Methods section. The localization of the fluorescence at the subhypostomal region is clearly shown in the longitudinal section representing the inset of picture D. Starvation enhances QR accumulation in the hypostomal region.

**Figure 9:**
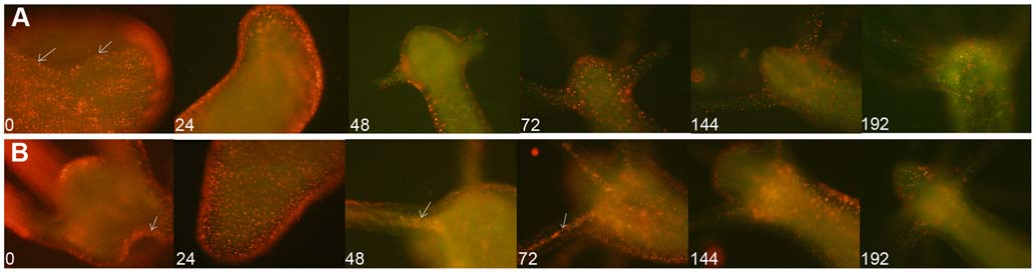
Dynamic of QR labelled cells during head and foot regeneration. Labelled polyps were bisected 24 h post treatment (time zero) and allowed to regenerate. At 24 h intervals, all specimens were monitored by fluorescence microscopy and imaged. A) Head regeneration. Picture at time t = 0 shows a decapitated body (the cutting region is indicated by the white arrows), which at 24 h shows the injured region completely closed. At 48 h the emerging tentacles appear labelled by QRs, which follow the ectodermal cell migration from the body to the tentacle tip, as tentacles grow (72 h, 144 h). At t = 192 h when the head regeneration is terminated, the QR fluorescence decreases. B) Foot regeneration. At time t = 0 the labelled polyp is bisected. QR labelled cells clearly show the cutting region (white arrow), which appears at t = 24 h completely closed. As regeneration process goes on, ecto- to endodermal migration of fluorescent material becomes progressively more evident, as indicated by the picture at times t = 48 h and t = 72 h. At this time, the tentacle endodermal localization of QRs become more evident (as indicated by the arrows), due to a corresponding fluorescence decrease at ectodermal level. At t = 144 h the fluorescence accumulates within the subhypostomal region and at t = 192 h it progressively decreases.

Thanks to the high brightness and photostability of the nanorods here synthesised we investigated the dynamic of QR uptake both over short and long periods, relative to the *Hydra* physiology. A time course performed over short incubation periods (10 to 30 min) showed 10 min p.i. that a very small number of mouth and tentacle cells had already engulfed the QRs in suspension (Figure 6A, 6B): the fluorescence appears dispersed within the cell cytoplasm. Later on, a process of QR packing occurred, as shown by the punctuated pattern of staining, illustrated by pictures taken 20 and 30 min p.i. (Figure 6C, 6D). QR uptake, initially restricted to tentacle battery cells, takes place also in the ectodermal cells lining the animal body, although at a lower extent and presenting variability, in term of total or partial body staining, among the specimens analysed (Figure S3). As this run on was performed on fixed specimens, the faster dynamic of internalization respect to the *in vivo* behaviour (shown in Figure 1A) might be due to the fixation procedures, and it confirms the differences between fixed and living cells observed at single cell level (see Figure 3). Cross- and longitudinal cryosections made from fixed treated specimens revealed that internalization is carried out by the ectodermal cell layer, facing directly the medium (Figure 7). Remarkably, 24 h post treatment, fluorescent material appears also into the endodermal cells lining the gastric cavity and the tentacles, while, at the tentacle base, the fluorescence draws a well defined strip along the tentacle length, shown by cross sections to be localised inside the endodermal cells and not in the tentacle lumen (Figure 7C, 7D; Figure S4). Culturing treated polyps both under normal feeding and starvation regimes revealed an intense accumulation of fluorescent particles within the subhypostomal region, often shaping a ring like structure, more pronounced in starved animals (Figure 8). The same dynamic was observed during regeneration of treated animals (Figure 9). While during head patterning (Figure 9A) QR labelled cells probe normal migration events [40], [41], during foot regeneration (Figure 9B) QR fluorescence is found at the tentacle base (about one third of the total length) in the inner endodermal layer, while the outer layer of the same region show decreased staining (Figure 9B, pictures at 48 h, 72 h). Although other vital dyes have been used in the past to study *Hydra* cell dynamics during differentiation [42], [43], development, regeneration [44] and interepithelial interaction [45], [46], this migration pattern, from ectoderm to endoderm, and the accumulation within the subhypostomal region, has not been described before.

### Uptake of Cadmium based QRs: effect on Hydra population growth

**Figure 10:**
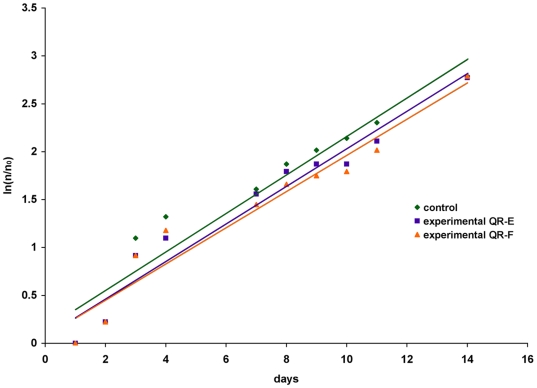
Influence of the QR treatment on Hydra population growth rate. Population growth test started with a population of four full-grown *Hydra*, incubated 4 h with 10 nM QR-E (violet), QR-F (orange), washed and equilibrated in culture solution or not treated (green). The individuals were inspected daily and counted under a stereomicroscope. There are not significant differences between treated and untreated population growth rates. The logarithmic growth rate constant (k) is the slope of the regression line using the the standard equation of logarithmic growth: ln(n/n_0_) = kt.

From dynamic and cell tracking studies, the presence of QRs in *Hydra* cells appeared not to affect cell survival or to interfere with animal physiology during developmental and regenerative processes. To measure the potential long-term toxic effects induced by QR labelling, we calculated the growth rates of QR treated polyps and compared them to untreated animals, under regular feeding regime. Growth rate of *Hydra* tissue is normally regulated by a balance between epithelial cell cycle length, phagocytosis of ectodermal cell in “excess”, and bud formation [47]. Thus, the population growth rate is an indirect measure of the *Hydra* tissue growth rate and cell viability. As shown in the graph of Figure 10 the growth rates between polyps treated with QRs (two different types, QR-E and QR-F were used) or untreated were similar, displaying similar budding rates and doubling population times (Table 3, Figure S5), indicating the absence of toxic effects. In order to estimate the amount of QRs uptaken by the animals, and to correlate the potentially toxic effect of the Cadmium (Cd) contained in the nanocrystal core to the animal survival, the intracellular Cd concentration was measured by means of elemental analysis. A Cd content equal to 0.33 and 0.54 mg/L, respectively after 4 and 24 h of incubation, was determined. These values are much lower compared to the median lethal concentration (LC50) value calculated for free Cd uptake (24 h LC50 = 4 mg/L), or to those causing minimal morphological alterations (3,2 mg/L), as reported [48], confirming the absence of toxicity of the Cd based QRs. This study indicate that the uptake process maximally occurs during the first hours of treatment and that incubation times of 4 h ensure a suitable amount of fluorescent nanocrystals available for cell labelling and tracking, not interfering with animal survival.

**Table 3:** Parameter of growth of *Hydra* treated with QRs.

Population	k	T_2_(days)	Budding rate (buds/Hydra/day)
Control (Not treated)	0.199	3.5	1.30
Experimental (QR-E)	0.203	3.4	1.31
Experimental (QR-F)	0.199	3.5	1.31

The growth constant k was calculated from experimental data as described in the Methods section. The *Hydra* population doubling time (T_2_) was obtained from the standard equation of logarithmic growth ln(n/n_0_) = kt. When n/n_0_ = 2. T_2_ = ln2/k = 0,693/k. The Average budding rate was obtained from total Budding Rate (BR) as BR/n_0_ (see also Figure S5). QR treatment does not influence the *Hydra* growth.

### QR uptake is prevented by annexin antibody

**Figure 11:**
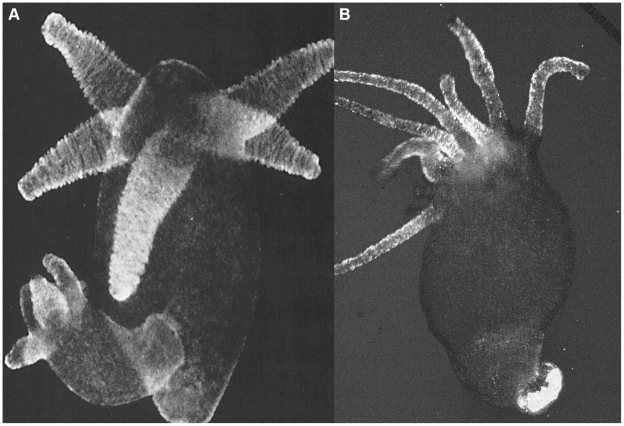
Comparison of ANX protein localization and QR labelling pattern. A) whole mount ANX immunolocalization using rabbit anti-ANX antibody, and detected using anti rabbit IgG alexa fluor 488 (adapted from [51] ). B) Living *Hydra* treated with fluorescent QRs. The overlapping of the two labelling patterns in the same cell types suggests the involvement of ANX in QR internalization.

Under mildly acidic conditions, *in vitro*, annexin XII (ANX) inserts into lipid membranes to form a transbilayer pore [49] and might be involved, *in vivo*, in the regulation of membrane trafficking [50]. In an attempt to investigate the molecular mechanism underlying QR uptake at acidic pH, living polyps were preincubated with anti-ANX antibody [51] 1 h before the addition of QRs to the culture medium. As shown in Table 4, affinity purified anti-ANX antibody completely inhibited QR uptake, even at very high dilution. To rule out the possibility that the inhibition was aspecifically due to the IgG, or to the BSA (bovin serum albumin) contained into the serum, the same assay was performed in presence of a not related antibody (human anti synaptic vesicle AP180) or of BSA, supplied at concentrations comparable to those of anti-ANX. Results showed that the inhibition of QR uptake was highly specific, and due to the presence of anti-ANX antibody, but not of other proteins. Thus, we provide functional evidence that the uptake of positively charged QRs is mediated by the presence of annexin on the membrane surface of ectodermal cells. This evidence is further supported by the fact that the immunofluorescence pattern of ANX in whole mount preparations [51] strikingly parallels the QR uptake pattern in the tentacle cells, the hypostome, and the peduncle cells, as illustrated in Figure 11.

**Table 4:** QR uptake is prevented by anti-ANX antibody.

Inhibition of QR uptake in presence of Anti-annexin XII	Inhibition of QR uptake in presence of anti AP-180	Inhibition of QR uptake in presence of BSA
1,25 µg (BSA 500 ng) +	5 µg (BSA 50 ng) −	5 µg −
500 ng (BSA200 ng) +	2 µg (BSA 20 ng) −	2 µg −
250 ng (BSA100 ng) +	1 µg (BSA 10 ng) −	1 µg −
125 ng (BSA 50 ng) +		500 ng −
25 ng (BSA10 ng) −		50 ng −

Groups of six polyps were equilibrated in SolHy pH 4 in presence of the indicated doses of the affinity purified antibodies (rabbit anti-annexin XII, rabbit anti human endocytic accessory protein AP180) or of bovin serum albumine (BSA). 1 h later QRs were added to the polyps and after at least 4 h of incubation the presence of internalization was monitored under a fluorescence microscope. As BSA is used for IgG stabilization, in each column is reported the amount of antibody used, together with the BSA co-administrated (in brackets). An additional set of experiment was performed by supplying pure BSA at different concentrations, to rule out its aspecific role in the uptake process. Inhibition of QR uptake, indicated by the plus sign (+) was observed in presence of anti-ANX, while was not prevented by either aspefic antibody nor BSA even at higher doses [indicated by the sign minus (−)].

## Discussion

Fluorescent semiconductor nanocrystals of different sizes, shapes, surface coatings, are increasingly being used in a wide range of biomedical applications, from cell biology to medical diagnostics. In spite of what has been achieved so far for targeting and tracking membrane proteins [52], [53], a complete understanding of how cells interact with nanostructures, at the molecular level, remains poorly understood. Here we show the feasibility to modulate endocytosis of amino-PEG coated CdSe/CdS QRs by engineering their surface chemistry and identified by a functional assay a membrane protein triggering their uptake.

**Figure 12:**
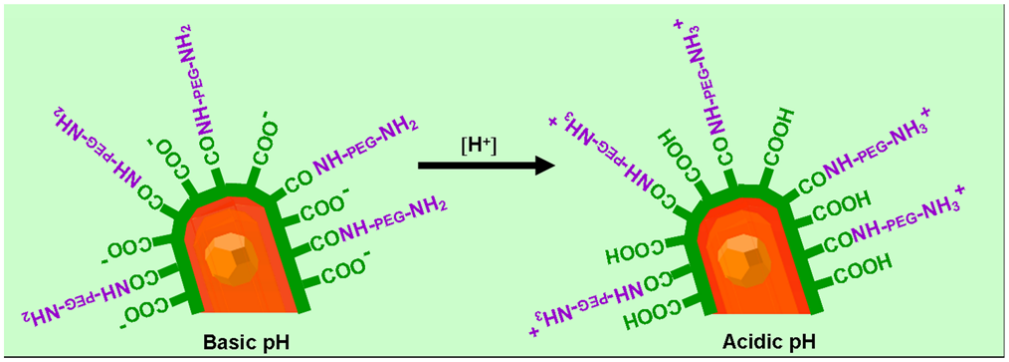
Protonation/de-protonation state of the QRs. A schematic view of the functional groups at the nanoparticle surface responsible for the switching of the surface charge. At basic pH, the carboxy groups are negatively charged and the amino groups are not protonated. At acidic pH, the carboxy and the amino groups are both protonated, which account for a positive zeta potential value measured. At neutral pH, the zeta potential measured in all cases is negative. This can be likely caused by the prevalence of non protonated carboxy groups (pka 4.0) of the polymer with respect to the positive charges of the amines at pH 7.

Modifying the amounts of amino-PEG molecules present on QR surface we were able to tune the net charges at the nanocrystal surface and thus the uptaking process. At acidic pH, the protonation of the PEG amino terminal groups (NH3^+^) contributes to increase the positive charges while the protonation of the carboxyl groups of the amphiphilic polymer shell causes a reduction of the negative charges (COO^−^) at the nanoparticle surface and indeed the sum of the two effects results in a net positive surface of the QR (Figure 12). The different amounts of PEG molecules attached at the same QR surface account for the different behaviours displayed by diverse nanorod samples, independently from their size and shape. QR presenting positive zeta potential bind to negatively charged membrane lipids, and stimulate endocytosis processes.

As the particles being internalized are nanometer-sized and suspended in the extracellular fluid, the uptake process should be termed pinocytosis [1]. However, while distinct size dependent mechanisms of pinocytosis have been described for endodermal digestive cells [54], similar knowledge for ectodermal cells is not available, and we will simply use the terms uptake/endocytosis to refer to this peculiar fluid-phase uptake. Technau and Holstein [43] reported on the phagocytosis of fluorescent polystyrene-based microspheres by epithelial cells. However, both the labelling kinetics and the spatial pattern of the labelled cells were profoundly different from those reported in this work. According to their method, 55% of all ectodermal cells were labelled by incubation in a 0.025% beads suspension for 15 h, while endodermal labelling was never detected, unless a 2.5% beads suspension was directly injected into the gastric cavity. Here, the incubation of *Hydra* in a suspension of fluorescent nanoparticles at nanomolar concentration resulted in the selective labelling of tentacle and hypostomal cells, as soon as 30 min post incubation. Not only the labelling efficiency and kinetics were different, but also the dynamic of labelled cells in cultured polyps. Our *in vivo* method for *Hydra* labelling highlighted new trafficking events between the two epithelial layers. The observed endodermal labelling from 24 h p.i. onward indicates a different behaviour for nanometer sized particles with regard not only to the endocytosis mechanism from the medium but also to their distribution to the other cells during animal growth. The mechanisms underlying this dynamic might be due to a multitude of events, from phagocytosis to migration of free nanoparticles, or of cells containing nanoparticles, from the ectoderm to the endoderm, and opens the way to future fascinating investigations. In *Hydra* phagocytosis has been involved in the regulation of cell number in response to feeding, in regeneration and in the removal of non–self cells [45], [47], [55], [56]. In response to cytostatic agents, ultrastructural images of endodermal cells engulfing dying interstitial cells have been captured [57], and very recently molecular evidences have shown the induction of autophagy both during starvation and regeneration [58]. Thus, in this primitive animal, in complete absence of mobile phagocytes, epithelial cells regulate their homeostasis and adapt to environmental conditions by phagocytosing apoptotic cells. As we also observed the endodermal staining by QRs enhanced during starvation and regeneration, an autophagy mechanism might be possibly underlying this phenomena, although other ongoing cell interactions should be provided to take into account for the progressive accumulation of fluorescent nanoparticles only at the subhypostomal level and at the tentacle base. The availability of markers for phagosome/autophagosome to use in biochemical and immunohystochemical analyses could help to address these issues [59].

Growth rates of polyps treated with two types of QRs did not show significant difference compared to untreated polyps, demonstrating the absence of QR toxic effects on the animal tissue growth, cell cycle length, reproductive capability. The low amount of free Cd measured by elemental analysis in crude homogenates of QR treated polyps if at one side accounts for the absence of toxicity, as elsewhere reported for similar doses [48], [60], at the other side highlights the feasibility to use these inorganic metal based nanoparticles, at nanomolar concentrations, as superior biological probes, with extraordinary brightness and stability. The low level of Cd found in animals exposed to QRs up to 24 h unabled us to carry on similar evaluations over longer periods, due both to the resolution limit of the instrument used for elemental analysis and to the progressive elimination of the QR labelled cells in growing animals.

In order to identify the cell molecular players regulating the uptake of positively charged QRs at acidic pH, our attention was caught by annexins, an evolutionary conserved multigene family with members being expressed throughout animal and plant kingdoms [30], [50], [61], [62]. Although the signature of the family is the Ca^2+^-dependent interaction with phospholipids through a conserved alpha-helical protein core domain [63], [64], ANXs can also undergo Ca^2+^-independent membrane interactions at mildly acidic pH [31], [65]–[67]. At pH 4.0 the helical hairpin protein domain assumes a transmembrane topography but, at pH approximately 5.0–5.5, it becomes peripheral, reversibly converted into the transmembrane form by lowering the pH. Moreover, in *Hydra*, ANX is expressed in the battery cells and in the hypostome, mirroring QR pattern (Figure 11). By treating living polyps with anti-ANX antibody prior to QR exposure, we were able to prevent QR uptake at acidic pH, supplying evidence, *in vivo*, for a crucial role of annexins in membrane trafficking events. We suggest that ANX mediates the interaction with positively charged QRs, organizing membrane domains and uptake processes, probably throughout the specie-specific amino terminal domain. In presence of anti-ANX antibody, the endocytosis machinery is blocked, probably due to impairment of functional or structural important ANX extracellular domains. While cellular and animal knock-out models have recently been established for a number of annexins, showing their participation in the regulation of membrane organization, membrane traffic, control of ion (Ca^2+^) currents across membranes [61], this is the first functional role found for *Hydra* ANX, *in vivo*. These knowledges will complement the structural and biophysical studies achieved using lipid vesicles, shading light on the functional role played by this peculiar protein.

In conclusion, by engineering nanoparticle surface charges, we were able to selectively control specific interactions between cell membrane and nanoparticles: the combined effect of pH dependent factors (QR positive charge and ANX membrane insertion) resulted in the active internalization of the nanorods in specific cell types and according to a precise temporal dynamic. As the uptake of nutrients and all communication among cells and between cells and their environment occurs through the plasma membrane, we provide new clues to control the cell portal of entry, and to understand the processes evoked at this interface by nanoscale objects with unique chemicophysical properties, which is a priority when designing nanodevices for biomedical purposes.

## Materials and Methods

### Synthesis and diamino-PEG functionalization of CdSe/CdS nanocrystals

CdSe/CdS nanorods (henceforward indicated as QRs) of different lengths and diameters were synthesized by a seeded-growth approach, as recently reported [32].

The surfactant-coated nanoparticles were made water-soluble by means of a polymer coating procedure: the polymer molecules formed an homogeneous and stable shell around the rods surface, where the outstretched carboxy groups allowed for the solubilization in mild basic solutions (sodium borate buffer, pH 9) through charge repulsion. The excess polymer was removed by an ultracentrifugation step[15]. Then, the carboxy moieties were used as anchoring units for the diamino-PEG molecules. The formation of an amide bond between one of the amino groups of the diamino-PEG and the carboxy group of one polymer unit was promoted by the addition of EDC (1-ethyl-3(3-dimethylaminopropyl)carbodiimide hydrochloride). The reaction occurred for 3 h at room temperature under vigorous stirring. The functionalization with amino-PEG increased the stability of the nanocrystals in a wide range of pH and ionic strength conditions. Furthermore, by varying the amount of diamino-PEG added it was possible to modulate the surface charge of the nanocrystals at different pH values, as reported in the zeta potentials curves of Figure 4 and Figure 5. Briefly, to each kind of nanocrystals, different ratios of EDC per QR were added into the solution mixture, as shown in Table S1 and Table S2.

At the end of the reaction, the QRs were washed several times on centrifuge filters (MWCO of 30,000 Dalton) in order to remove the unbound diamino-PEG. The last washing step was performed with milliQ water.

Low resolution TEM images of the amino-PEG functionalized nanocrystals were recorded with a Jeol Jem 1011 microscope operating at an accelerating voltage of 100 kV.

Zeta potential measurements were performed on a Zetasizer Nano ZS90 (Malvern) equipped with a 4.0 mW He-Ne laser, operating at 633 nm, and an avalanche photodiode detector. Prior to the measurements, the samples were dissolved in solutions at different pH values.

UV-vis absorption spectra and Photoluminescence (PL) spectra were recorded on a Varian Cary 300 UV-Vis spectrophotometer. and on a Cary Eclipse spectrophotometer, respectively.

The electrophoretic characterization of the samples was carried out on 2% agarose gel at 100 V for 1 h on a Biorad system. Prior to gel electrophoresis, to each sample a solution corresponding to 20% of the sample volume and containing Orange G and 30% glycerol in loading buffer was added. After the run the gel was observed under UV light.

### Elemental analysis

One thousand polyps were incubated 4 and 24 h with 10 nM QR-A, washed, digested by the addition of a HCl/HNO3 3∶1 (v/v) solution and the intracellular Cd concentration was measured by means of ICP-AES (Inductively Coupled Plasma Atomic Emission Spectrometer).

### Hydra culture

*Hydra vulgaris* (strain Zurich, originally obtained by P.Tardent) were asexually cultured in physiological solution (SolHy: 1 mM CaCl_2_, 0.1 mM NaHCO_3_, pH 7) by the method of Loomis and Lenhoff with minor modifications [68]. The animals were kept at 18±1°C and fed three times per week with freshly hatched *Artemia salina nauplii*. Polyps from homogeneous populations, three-weeks-old and carrying one or two buds, were selected for the experiments.

### In vivo experiments with intact and regenerating animals

Groups of 10 animals were collected in plastic multiwells, allowed to equilibrate at room temperature in 300 µl of physiological solution (SolHy) buffered with 1 mM Tris HCl (pH 7, or pH 4, as indicated). The test was initiated by adding 10 nM CdSe/CdS core/shell QRs to each well containing 10 polyps and incubating for periods ranging from 10 minutes to 4 h. QR uptake was monitored *in vivo*, unless otherwise stated, by continuous videorecording using a Camedia-digital camera (Olympus) connected to a stereomicroscope (Olympus ZSX-RFL2) equipped with fluorescence filter sets (BP460–490/DM505/LP510). Following extensive washes, *in vivo* imaging was accomplished at several magnification by using both a stereomicroscope and an inverted microscope (Axiovert 100, Ziess) equipped with a digital colour camera (Olympus, DP70) and fluorescence filter sets (BP450–490/FT510/LP515). For imaging acquisition and analysis the software system Cell F (Olympus) was used. The involvement of Ca^2+^ ions in the uptaking process was evaluated by performing experiments in 300 µl of Ca^2+^ free medium, namely in 0.1 mM NaHCO_3_, buffered with 1 mM Tris HCl (pH 7 or pH 4) or in SolHy added of 4 mM EGTA as Ca^2+^ chelator. The time course over short periods (run on from 10 to 30 mintues) was performed by incubating the animals for the indicated times, washing with clean SolHy, relaxation for 2 min in 2% urethan, fixation with 4% paraformaldeide for 16 h, and whole mount microscopy observation.

For regeneration experiments, QR treated polyps were bisected in the gastric region and *in vivo* imaged at various time points post amputation.

Experiments were performed in air-conditioned environment at 22°C, and repeated three times for each condition tested.

### Hydra growth and budding rates

Experimental animals (four *Hydra* with one bud) were treated with the indicated QR (either QR-E R1 or QR-F R1), for 4 h, then washed, and the following day placed in 3,5 cm petri dishes (1 *Hydra*/dish). Control animals at the same developmental stage were not treated. Both experimental and control *Hydra* were fed once daily and the following two growth parameters were determined: 1) Population doubling time. The growth rate costant (k) of an exponentially growing group of animals is defined as *ln(n/n_0_) = kt* where n is the number of animals at time t and n_0_ the number of animal at t_0_. For n/n_0_ = 2, t = T_2_, the doubling time of the population. T_2_ was determined by linear regression [47]. 2) Budding rate. The budding rate was determined as the average number of buds produced per *Hydra* per day. Experimentally the increase of buds per *Hydra* was counted daily.

### Hydra tissue manipulation and cryosectioning

*Hydra* polyps were dissociated according to Flick and Bode [35] with minor modifications. This procedure allows to dissociate *Hydra* tissue into a suspension of viable single cells. Briefly, test animals were rinsed with SolHy and soaked in the dissociation solution for 60 minutes at 0°C. Following a mechanic dissociation by a glass narrowed pipette, the suspension containing dissociated cells were transferred on microscope slides for fluorescence microscopy.

For analysis of cell types labelled by QRs whole animals were macerated into a suspension of fixed single cells as described [36]. Briefly, whole animals were macerated in a solution containing glycerol/acetic acid/water (1∶1∶13) for 20 min on ice and fixed by the addition of paraformaldehyde (2% wt/vol). The cell suspension was spread onto gelatine coated slides (Superfrost microscope slides, Menzel) and allowed to air dry overnight. The cells attached to the glass slides were observed by optical merging between phase-contrast and fluorescence imaging.

For tissue sectioning, test animals were fixed in 4% paraformaldeyde pH 7.4, at 4°C, rinsed three times in phosphate saline buffers (PBS: 8 g/l NaCl; 0.2 g/l KCl; 1.44 g/l Na_2_HPO_4_•7H_2_0; 0.24 g/l KH_2_PO_4_), soaked over night in 30% saccarose in PBS and then embedded in the frozen section medium Neg-50 (Richard-Allan Scientific). Cryo-sections of 10 µm thickness were obtained by a cryostat (Leitz, digital 1760), collected on gelatine coated slides (Superfrost microscope slides, Menzel) and mounted in Aquatex mounting medium (Merk) before imaging.

### Antibody exposure

The effect of specific and aspecific antibodies on the modulation of QR uptake was evaluated by adding the affinity purified antibodies or pure bovin serum albumine (BSA, Sigma) to groups of six *Hydra* bathed in SolHy at pH 4. 1 h post incubation, 10 nM QRs were added to each well, and QR uptake monitored by fluorescence imaging in *vivo*, as above described. Antibodies used: the polyclonal rabbit anti-*Hydra* ANX was a kind gift of Dr. X.Zhang, University of Kansas Medical Center, Kansas City, Kansas) [51]; polyclonal rabbit Anti AP-180 (human endocytic assembling protein AP-180) [69] was gifted from SYSY (Synaptic System, Germany).

## Supporting Information

Figure S1 Characterization of the samples QR-A, QR-B, QR-C, QR-D. A) Sketches showing the structure and the size of the samples used: the inorganic core (shown in yellow) is coated by an organic layer made of polymer and diamino-PEG (drawn as a green shell). B) TEM images of the water-soluble QRs (the scale bar corresponds to 50 nm). C) UV-vis absorption and photoluminescence spectra of the diamino-PEG functionalized QRs.
(2.66 MB TIF)

Figure S2 Characterization of the samples QR-E and QR-F. A) Sketches showing the structure and the size of the samples used: the inorganic core (pink) is coated by an organic layer made of polymer and diamino-PEG (drawn as a green shell). B) TEM images of the water-soluble QRs (the scale bar corresponds to 50 nm). C) UV-vis absorption and photoluminescence spectra of the diamino-PEG functionalized QRs. D) gel electrophoresis of the polymer coated and the diamino-PEG QRs. The label R1 refers to the sample functionalized with higher amount of amino-PEG, while R2 refers to the same QR sample functionalized with less amount of amino-PEG.
(1.50 MB TIF)

Figure S3 Pattern of QR labelling in different animals. Challenging living Hydra with QRs resulted in nanoparticle uptake by cells surrounding the hypostomal tip, tentacle battery cells and at a lower extent by ectodermal cells along the gastric region and the peduncles. Althought 90% of the polyps treated for 2 h with QRs show selective uptaking in the tentacle and hypostomal regions, the 10% showed QR fluorescence all over the body (i.e. top right panel), indicating the capability of ectodermal cells to uptake the nanoparticles, but with a different affinity. Experiments were performed on n = 100 polyps. Scale bar = 1 mm.
(7.06 MB TIF)

Figure S4 Tracking QR fluorescence on tissue sections. Polyps were incubated with QRs for 2 h, at pH 4, extensively washed, and cultured in SolHy at physiological pH for 4 h, 48 h and 72 h. Tissue sections, obtained as described in the Materials and Methods, were imaged by fluorescence microscopy. A) Longitudinal sections show that QRs, initially located in the ectoderm, at 48 h are found into the endoderm layer (white arrow). The section at 72 h shows most of QR containing cells at the tentacle tip, where cell displacement occurs, while in the central part they are located only into the endodermal layer. The insets show cross sections at the levels indicated by the white dotted lines, indicating that the QR location in the central part of the tentacle is inside endodermal cells and not into the tentacle lumen while at tip level the location is within the ectodermal cells. B) Fluorescence and bright field optical merge imaging of Hydra tissue sections, counterstained with toluidine blue. On the left panel is shown a cross section of a polyp treated 4 h with QRs. Fluorescence is located in the apical part of the ectodermal cells. On the right panel a cross section at level of the gastric cavity, showing endodermal staining 48 h post treatment. Ec = ectoderm; en = endoderm; m = mesoglea.
(1.80 MB TIF)

Figure S5 Budding rate of Hydra populations. For each experimental condition n0 = 4 full-grown Hydra, were incubated for 4 h with 10 nM QR-E (violet squares), QR-F (orange triangles), washed and equilibrated in culture solution or not treated (green rhombi). The individuals were inspected daily and counted under a stereomicroscope. Total detached buds against time (in days) are reported. The budding rate of the three populations was calculated from the slope of the regression lines (graph black lines). The total Budding Rates (BR) of treated and untreated population are similar. Average budding rates, calculated as BR/n0, are reported in Table 3.
(0.10 MB TIF)

Table S1 Conditions for the diamino PEG reaction for the preparation of the QR-A, QR-B, QR-C, and QR-D. Column 1: QR concentration; columns 2 and 3, respectively, ratios of diamino-PEG and EDC per nanoparticle (NP) used.
(0.03 MB DOC)

Table S2 Conditions for the diamino PEG reaction for the preparation of the QR-E R1, QR-E R2, QR-F R1 and QR-F R2. Column 1: QR concentration; columns 2 and 3, respectively, ratios of diamino-PEG and EDC per nanoparticle (NP) used.
(0.03 MB DOC)
