# Supplementary figures and images for: Fluorescent Nanocrystals Reveal Regulated Portals of Entry into and Between the Cells of Hydra
Source: PLoS One. 2009 Nov 2;4(11):e7698. doi: 10.1371/journal.pone.0007698 (PMC2765617; doi:10.1371/journal.pone.0007698)

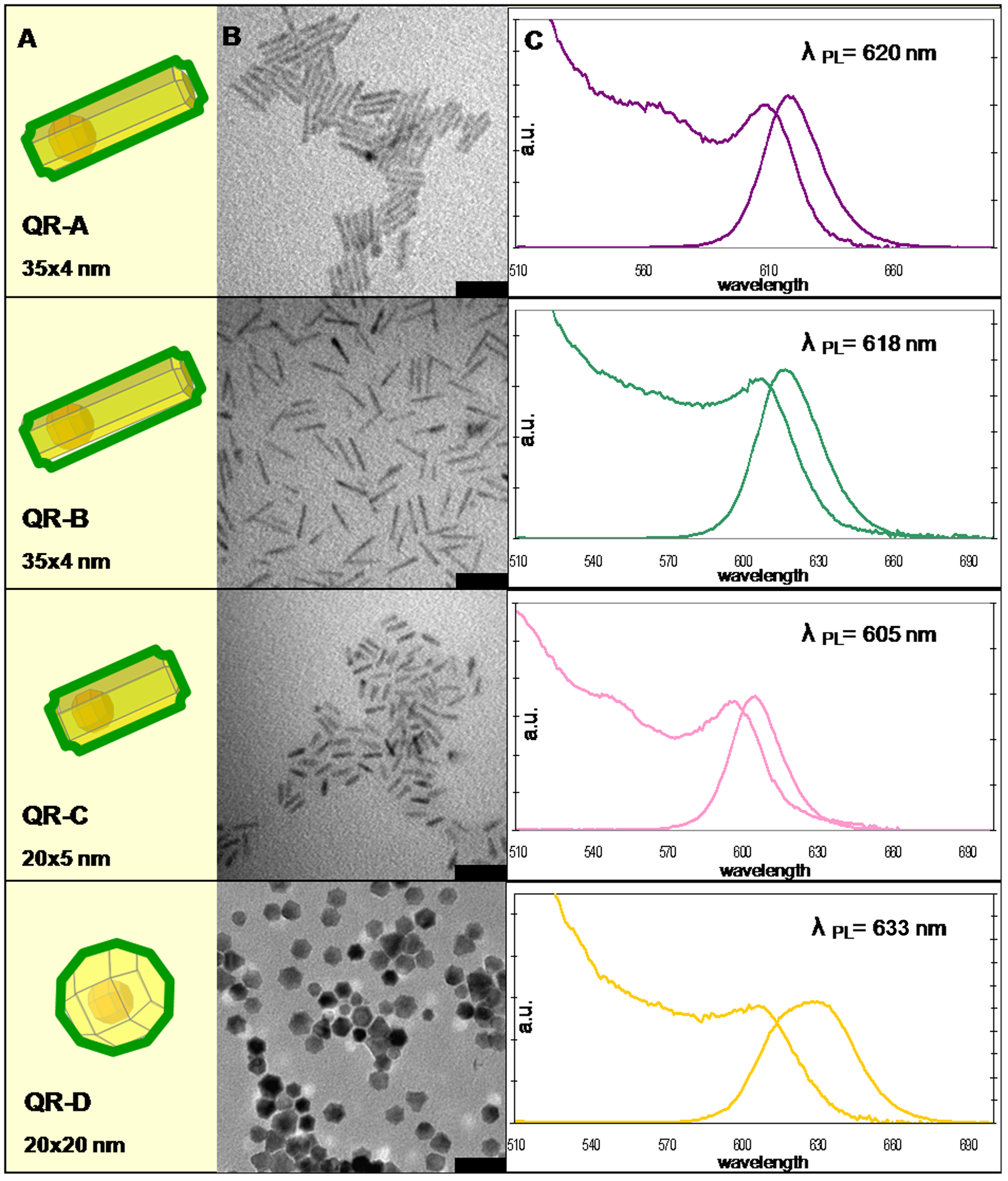

Supplement: Figure S1 — Characterization of the samples QR-A, QR-B, QR-C, QR-D. A) Sketches showing the structure and the size of the samples used: the inorganic core (shown in yellow) is coated by an organic layer made of polymer and diamino-PEG (drawn as a green shell). B) TEM images of the water-soluble QRs (the scale bar corresponds to 50 nm). C) UV-vis absorption and photoluminescence spectra of the diamino-PEG functionalized QRs. (2.66 MB TIF) [file pone.0007698.s001.tif]

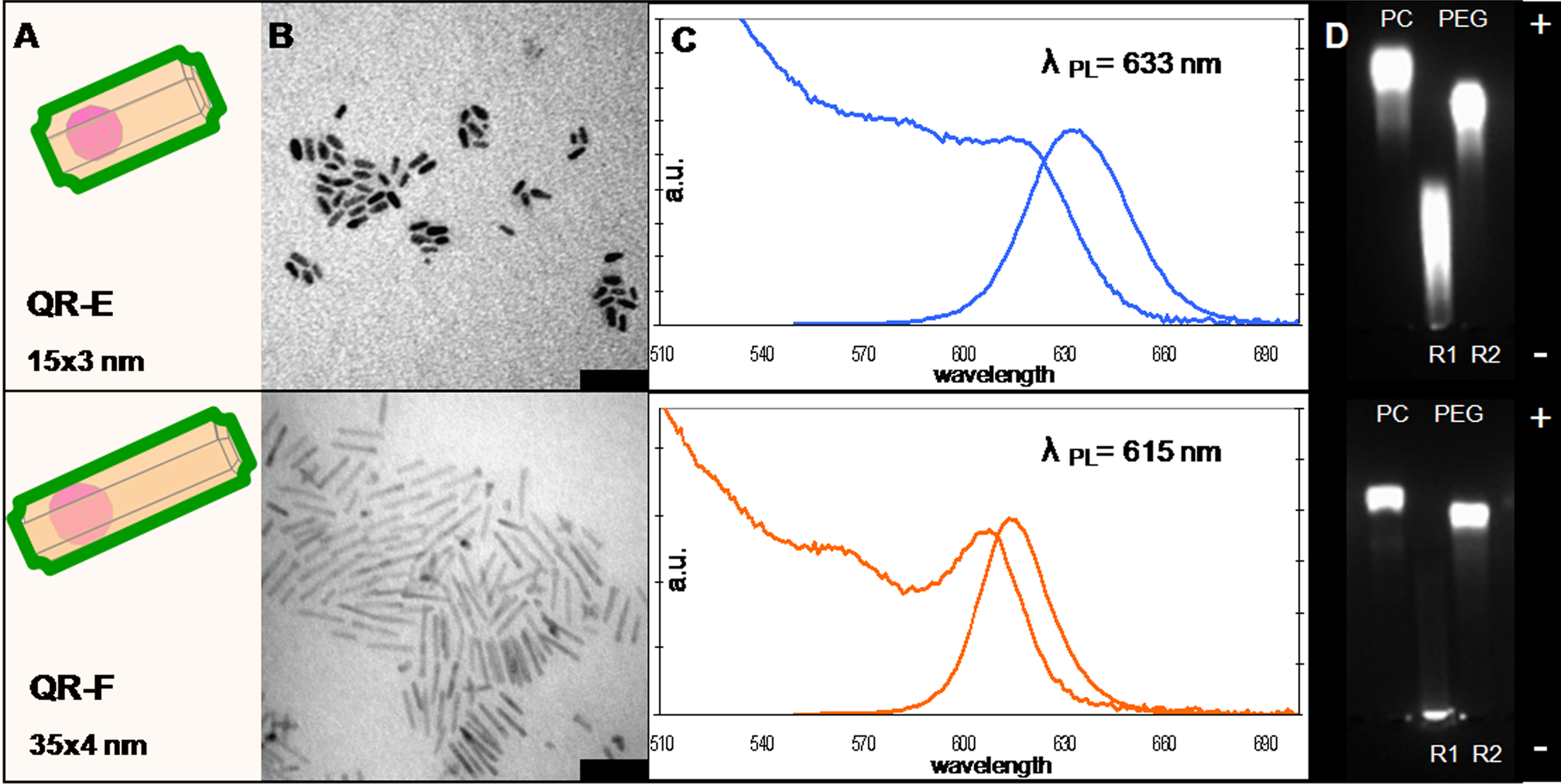

Supplement: Figure S2 — Characterization of the samples QR-E and QR-F. A) Sketches showing the structure and the size of the samples used: the inorganic core (pink) is coated by an organic layer made of polymer and diamino-PEG (drawn as a green shell). B) TEM images of the water-soluble QRs (the scale bar corresponds to 50 nm). C) UV-vis absorption and photoluminescence spectra of the diamino-PEG functionalized QRs. D) gel electrophoresis of the polymer coated and the diamino-PEG QRs. The label R1 refers to the sample functionalized with higher amount of amino-PEG, while R2 refers to the same QR sample functionalized with less amount of amino-PEG. (1.50 MB TIF) [file pone.0007698.s002.tif]

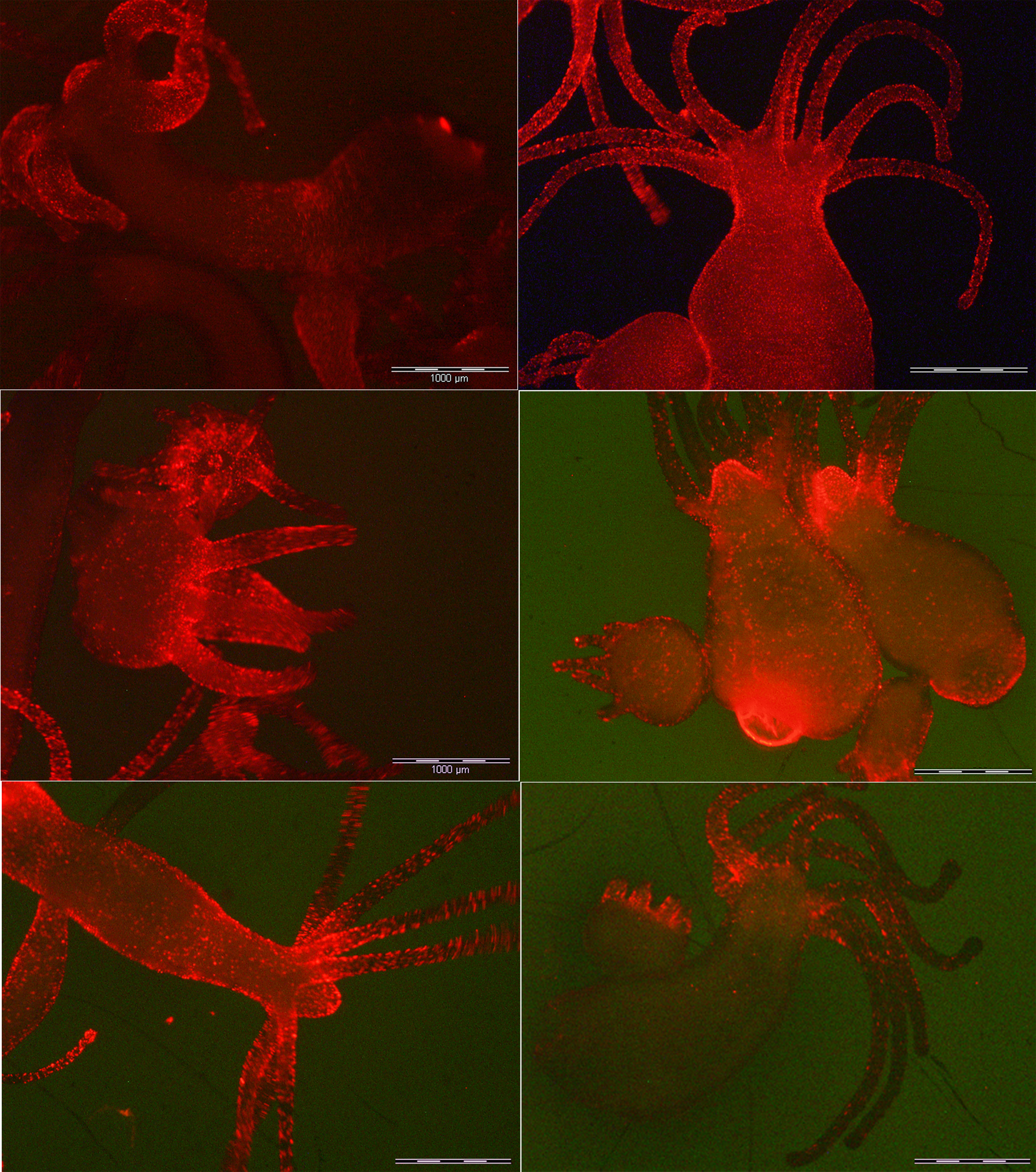

Supplement: Figure S3 — Pattern of QR labelling in different animals. Challenging living Hydra with QRs resulted in nanoparticle uptake by cells surrounding the hypostomal tip, tentacle battery cells and at a lower extent by ectodermal cells along the gastric region and the peduncles. Althought 90% of the polyps treated for 2 h with QRs show selective uptaking in the tentacle and hypostomal regions, the 10% showed QR fluorescence all over the body (i.e. top right panel), indicating the capability of ectodermal cells to uptake the nanoparticles, but with a different affinity. Experiments were performed on n = 100 polyps. Scale bar = 1 mm. (7.06 MB TIF) [file pone.0007698.s003.tif]

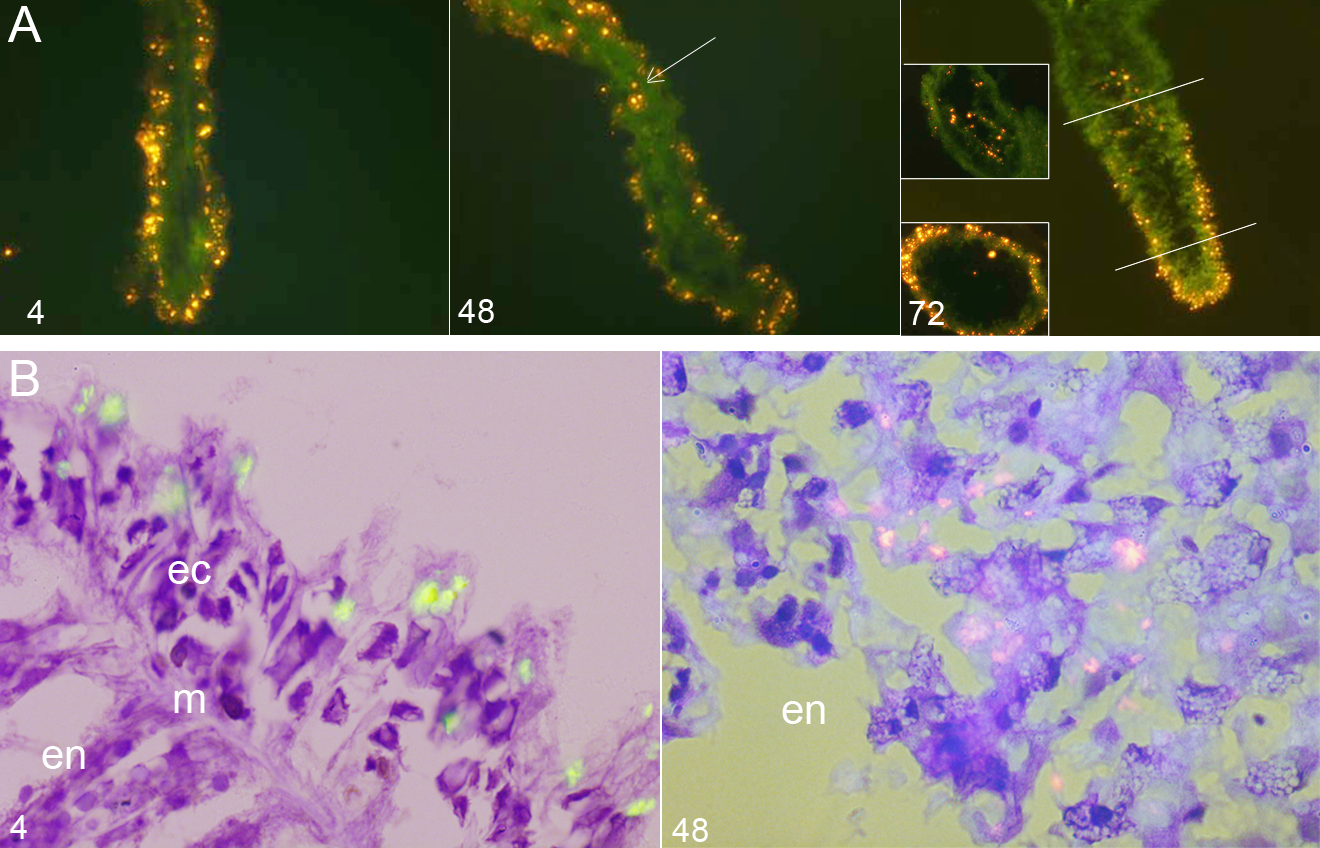

Supplement: Figure S4 — Tracking QR fluorescence on tissue sections. Polyps were incubated with QRs for 2 h, at pH 4, extensively washed, and cultured in SolHy at physiological pH for 4 h, 48 h and 72 h. Tissue sections, obtained as described in the Materials and Methods, were imaged by fluorescence microscopy. A) Longitudinal sections show that QRs, initially located in the ectoderm, at 48 h are found into the endoderm layer (white arrow). The section at 72 h shows most of QR containing cells at the tentacle tip, where cell displacement occurs, while in the central part they are located only into the endodermal layer. The insets show cross sections at the levels indicated by the white dotted lines, indicating that the QR location in the central part of the tentacle is inside endodermal cells and not into the tentacle lumen while at tip level the location is within the ectodermal cells. B) Fluorescence and bright field optical merge imaging of Hydra tissue sections, counterstained with toluidine blue. On the left panel is shown a cross section of a polyp treated 4 h with QRs. Fluorescence is located in the apical part of the ectodermal cells. On the right panel a cross section at level of the gastric cavity, showing endodermal staining 48 h post treatment. Ec = ectoderm; en = endoderm; m = mesoglea. (1.80 MB TIF) [file pone.0007698.s004.tif]

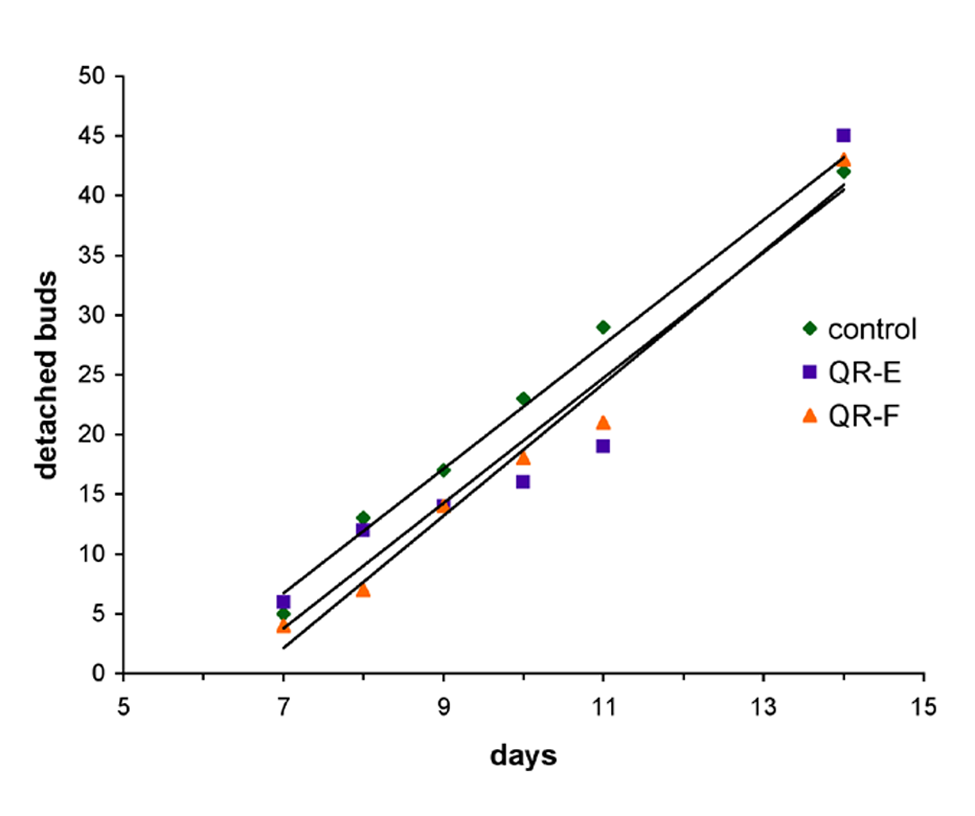

Supplement: Figure S5 — Budding rate of Hydra populations. For each experimental condition n0 = 4 full-grown Hydra, were incubated for 4 h with 10 nM QR-E (violet squares), QR-F (orange triangles), washed and equilibrated in culture solution or not treated (green rhombi). The individuals were inspected daily and counted under a stereomicroscope. Total detached buds against time (in days) are reported. The budding rate of the three populations was calculated from the slope of the regression lines (graph black lines). The total Budding Rates (BR) of treated and untreated population are similar. Average budding rates, calculated as BR/n0, are reported in Table 3. (0.10 MB TIF) [file pone.0007698.s005.tif]
