# Supplementary material for: Fluorescent Nanocrystals Reveal Regulated Portals of Entry into and Between the Cells of Hydra
Source: PLoS One. 2009 Nov 2;4(11):e7698. doi: 10.1371/journal.pone.0007698 (PMC2765617; doi:10.1371/journal.pone.0007698)
Supplement: Table S1 — Conditions for the diamino PEG reaction for the preparation of the QR-A, QR-B, QR-C, and QR-D. Column 1: QR concentration; columns 2 and 3, respectively, ratios of diamino-PEG and EDC per nanoparticle (NP) used. (0.03 MB DOC) [file pone.0007698.s006.doc]

|  | [NP] | r PEG:NP | r EDC:NP |
| --- | --- | --- | --- |
| QR-A | 1 x 10^-6^ M | 10^3^ : 1 | 10^5^ : 1 |
| QR-B | 1 x 10^-6^ M | 10^3^ : 1 | 10^5^ : 1 |
| QR-C | 1 x 10^-6^ M | 10^3^ : 1 | 10^5^ : 1 |
| QR-D | 1 x 10^-6^ M | 10^3^ : 1 | (6.5 x 10^4^) : 1 |

**Table S1.** Conditions for the diamino PEG reaction for the preparation of the QR-A, QR-B, QR-C, and QR-D. Column 1: QR concentration; columns 2 and 3, respectively, ratios of diamino-PEG and EDC per nanoparticle (NP) used.
