# Supplementary material for: Fluorescent Nanocrystals Reveal Regulated Portals of Entry into and Between the Cells of Hydra
Source: PLoS One. 2009 Nov 2;4(11):e7698. doi: 10.1371/journal.pone.0007698 (PMC2765617; doi:10.1371/journal.pone.0007698)
Supplement: Table S2 — Conditions for the diamino PEG reaction for the preparation of the QR-E R1, QR-E R2, QR-F R1 and QR-F R2. Column 1: QR concentration; columns 2 and 3, respectively, ratios of diamino-PEG and EDC per nanoparticle (NP) used. (0.03 MB DOC) [file pone.0007698.s007.doc]

|  | [NP] | r PEG:NP | r EDC:NP |
| --- | --- | --- | --- |
| QR-E-R1 | 1 x 10^-6^ M | (5 x 10^2^) : 1 | 10^6^ : 1 |
| QR-E-R2 | 1 x 10^-6^ M | (5 x 10^2^) : 1 | (6 x 10^4^) : 1 |
| QR-F-R1 | 1 x 10^-6^ M | (5 x 10^2^) : 1 | (2.5 x 10^5^) : 1 |
| QR-F-R2 | 1 x 10^-6^ M | (5 x 10^2^) : 1 | (3 x 10^4^) : 1 |

**Table S2. Conditions for the diamino PEG reaction for the preparation of the QR-E R1, QR-E R2, QR-F R1 and QR-F R2.** Column 1: QR concentration; columns 2 and 3, respectively, ratios of diamino-PEG and EDC per nanoparticle (NP) used.
